# Long-Term Follow-Up, Treatment Strategies, Functional Outcome, and Health-Related Quality of Life after Surgery for WHO Grade 2 and 3 Intracranial Meningiomas

**DOI:** 10.3390/cancers14205038

**Published:** 2022-10-14

**Authors:** Jenny Pettersson-Segerlind, Alexander Fletcher-Sandersjöö, Ann-Christin von Vogelsang, Oscar Persson, Lars Kihlström Burenstam Linder, Petter Förander, Tiit Mathiesen, Erik Edström, Adrian Elmi-Terander

**Affiliations:** 1 Department of Neurosurgery, Karolinska University Hospital, 171 64 Stockholm, Sweden; 2 Department of Clinical Neuroscience, Karolinska Institutet, 171 77 Stockholm, Sweden; 3 Department of Neurosurgery, Rigshospitalet, Institute of Clinical Medicine, University of Copenhagen, 2100 Copenhagen, Denmark

**Keywords:** meningioma, WHO grade 2, WHO grade 3, neurosurgery, health-related quality of life, patient-reported outcomes, return to work, gamma-knife radiosurgery, radiotherapy, chemotherapy

## Abstract

**Simple Summary:**

Meningiomas are the most common group of primary intracranial tumors. While the majority are classified as WHO grade 1, WHO grade 2 and 3 meningiomas have poorer outcomes, even after gross total resection, and often require supplementary treatment. Long-term follow-up data regarding the progression-free survival (PFS) and overall survival (OS) for grade 2 and 3 tumors are scarce, and data evaluating the routine use of supplementary radiotherapy and radiosurgery have been inconclusive. Furthermore, few studies have reported data on the health-related quality of life (HRQoL), anxiety, and depression for these patients. In this population-based cohort study, we reviewed 51 cases of WHO grade 2 and 3 meningiomas. We found that the median OS was 13 years for grade 2 and 1.4 years for grade 3 meningiomas. Meningioma was the cause of death in 93% of the patients who passed away. The surviving patients showed HRQoL measures comparable to that of the general population, with the exception of significantly more anxiety and depression. All patients who worked preoperatively returned to work after their treatment.

**Abstract:**

Progression-free survival (PFS) and overall survival (OS) for WHO grade 2 and 3 intracranial meningiomas are poorly described, and long-term results and data evaluating the routine use of supplementary fractionated radiotherapy (RT) or stereotactic radiosurgery (SRS) has been inconclusive. The aim of this study was to determine the long-term PFS and OS at a center that does not employ routine adjuvant RT. For this purpose, a retrospective population-based cohort study was conducted of all WHO grade 2 and 3 meningiomas surgically treated between 2005 and 2013. The cohort was uniformly defined according to the WHO 2007 criteria to allow comparisons to previously published reports. Patient records were reviewed, and patients were then prospectively contacted for structured quality-of-life assessments. In total, 51 consecutive patients were included, of whom 43 were WHO grade 2 and 8 were grade 3. A Simpson grade 1–2 resection was achieved in 62%. The median PFS was 31 months for grade 2 tumors, and 3.4 months for grade 3. The median OS was 13 years for grade 2, and 1.4 years for grade 3. The MIB-1-index was significantly associated with an increased risk for recurrence (*p* = 0.018, OR 1.12). The median PFS was significantly shorter for high-risk tumors compared to the low-risk group (10 vs. 46 months; *p* = 0.018). The surviving meningioma patients showed HRQoL measures comparable to that of the general population, with the exception of significantly more anxiety and depression. All patients who worked before surgery returned to work after their treatment. In conclusion, we confirm dismal prognoses in patients with grade 2 and 3 meningiomas, with tumor-related deaths resulting in severely reduced OS. However, the cohort was heterogenous, and a large subgroup of both grade 2 and 3 meningiomas was alive at 10 years follow-up, suggesting that a cure is possible. In addition, fractionated radiotherapy and chemotherapy had little benefit when introduced for recurrent and progressive diseases.

## 1. Introduction

Meningiomas are the most common intracranial tumors, accounting for 30–35%. In comparison, glioblastomas account for 15%, and gliomas of any grade account for 28% [1]. Most meningiomas are benign and classified as World Health Organization (WHO) grade 1. However, approximately 5–34% are atypical (WHO grade 2) and 1–3% are malignant/anaplastic (WHO grade 3) [1,2]. WHO grade 2 and 3 meningiomas have poorer outcomes, even after gross total resection, and often require supplementary treatment such as fractionated radiotherapy (RT), stereotactic radiosurgery (SRS), and chemotherapy [3,4]. The poor outcomes and the complexity of the management of higher-grade meningiomas have resulted in the evolution of different management strategies, even though surgery remains the mainstay of treatment.

This single-center cohort study aimed to evaluate and summarize the long-term clinical courses for patients with grade 2 and 3 meningiomas. In the first part, outcomes of surgical and nonsurgical treatments are evaluated, and in the second part, we provide an in-depth evaluation of HRQoL-measures in this patient group.

## 2. Part 1, Long-Term Follow-Up and Outcome

### 2.1. Introduction

The definitions of grade 2 and 3 meningiomas have changed with every version of the WHO grading system [2,5,6,7,8]. With the adoption of the 2021 version, the incidence of grade 2 and 3 meningiomas increased from <10% to 35%, with WHO grade 2 tumors accounting for most of this increase [9]. The 2016 version of the WHO classification scheme was the first to include specific molecular alterations in the diagnoses of tumors. The 2021 classification lists 15 distinct morphologic meningioma subtypes that range in WHO grade from 1 to 3 [6]. Despite several studies characterizing the molecular alterations in meningiomas, they do not impact the classification with two exceptions: TERT promoter mutations and the homozygous deletion of CDKN2A/B have been associated with a more aggressive clinical behavior and their presence suffices for a grade 3 classification even if the histopathology does not meet the criteria [10,11]. Consequently, data and treatment recommendations based on previous versions of the WHO grading system may lack precision and be difficult to implement. To resolve this difficulty, the definition of a high-risk category, which takes into account both the WHO grade and the extent of surgical resection, has been proposed. High-risk patients are those with a new or recurring WHO grade 3 meningioma of any resection extent, recurrent WHO grade 2 of any extent of resection, or new WHO grade 2 after subtotal resection (Simpson grade 3 or higher) [12].

The treatment recommendations for grade 2 and 3 meningiomas frequently include conventional fractionated RT and intensified surveillance for recurrences. Several studies have sought to understand the benefit of RT [12,13,14]. However, the radiation modality and the treatment doses are still a matter of debate, and reproducible data to support guidelines are scarce [15,16,17]. Moreover, the clinical course for grade 3 meningiomas is highly variable [18], and the evidence in support of routine adjuvant radiotherapy for grade 3 meningiomas is inconclusive [19]. It has been reported that the 3-year progression-free survival (PFS) for high-risk meningiomas after adjuvant RT is 59%, with 69% local control and 79% overall survival (OS) [12]. Kent et al. reported that the addition of adjuvant RT to surgery significantly increased the 3-year PFS from 39% to 66% [14].

SRS is an important adjunct to the management of intracranial low-grade meningiomas, with low treatment-related morbidity and reported long-term (>5 y) tumor control rates ranging from 86–100% [20,21,22]. Reported outcomes after SRS in grade 2 and 3 meningiomas are limited to observational studies. The 5-year progression-free survival is between 35% and 40%, while the 5-year overall survival is 75–80% [23,24].

Due to the lack of proven efficacy, chemotherapeutics do not have an established role in the treatment of meningiomas, and current recommendations suggest chemotherapy for progressive disease only when all other options have been exhausted [25]. At the study center, temozolomide, a DNA-alkylating agent, or Bevacizumab, an angiogenesis inhibitor, are used for rescue therapy. However, their utility, especially in high-grade meningiomas, is unclear [26,27].

To shed light on these questions and improve external validity, this study aims to describe the long-term (>10 years) clinical courses, surgical and nonsurgical treatments, PFS and OS, and patient-reported outcomes in a cohort of grade 2 and 3 meningiomas, as classified according to the WHO 2007 classification.

### 2.2. Materials and Methods

#### 2.2.1. Patient Selection and Study Setting

All adult patients (≥18 years) who were surgically treated for a grade 2 or 3 meningioma at the study hospital between 2005–2013 were eligible for inclusion. The study hospital is a publicly funded and owned tertiary care center serving a region of roughly 2.3 million inhabitants, the only neurosurgical center in the region, and the only center operating a Gamma-knife in the country. Patients were identified using the surgical management software Orbit (Evry Healthcare Systems, Solna, Sweden). Medical records and imaging data from digital hospital charts were retrospectively reviewed using the health record software TakeCare (CompuGroup Medical Sweden AB, Farsta, Sweden).

#### 2.2.2. Surgical Technique and Follow-Up Routine

The details of the surgical technique are outside the scope of this manuscript. At the study center, patients with WHO grade 1 meningioma are followed with an outpatient visit at 3 months and routine MRI at 3 months, and 1, 3, 5, and 10 years after surgery. When a diagnosis of a WHO grade 2 or 3 meningiomas is made, radiological follow-ups are performed annually instead. In cases with residual tumors or recurrences, the patients are discussed at multidisciplinary conferences, where neurosurgeons, neuroradiologists, and neuro-oncologists participate. At the conferences, individual arrangements for follow-up and treatments, either surgical or supplementary radio- or chemotherapy, are discussed. In these cases, the patients are offered multiple outpatient visits or telephone contacts with members of their treatment team.

#### 2.2.3. Variables Retrieved from Electronic Medical Records

Baseline data included demographic parameters, imaging findings, treatment data, tumor characteristics, and clinical parameters such as the Karnofsky Performance Status (KPS). Tumor growth was defined as the radiological growth of a tumor remnant following subtotal resection, while tumor recurrence was defined as the appearance of a new meningioma following gross total resection. To evaluate the effect of the number of treatments that a patient was subjected to during the course of the disease, the sum of surgeries, gamma-knife treatments, RT, and chemotherapy was calculated. In this context, a course of RT was counted once as was chemotherapy, which was typically given continuously if effective.

While 15 patients had undergone their first meningioma resection prior to the study period, PFS and OS were calculated from the first operation performed during the study period (2005–2013). This was done because (i) the WHO grade was often uncertain for procedures performed before 2005, and (ii) not doing so would bias the data, as we were not able to include previously operated patients who did not undergo renewed surgery during the study period.

All histopathological analyses were performed at the Department of Pathology, Karolinska University Hospital, Stockholm, Sweden. The MIB-1-indexes were analyzed manually. Patients were classified according to WHO criteria from 2007.

#### 2.2.4. Patient Selection

**Figure 1:**
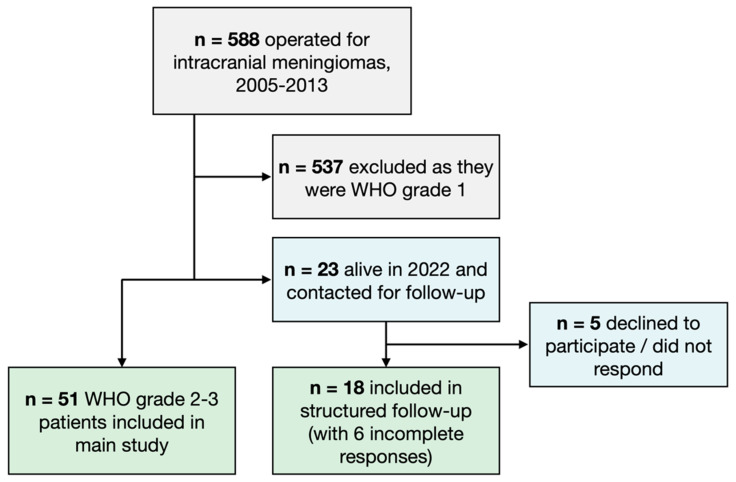
Flowchart showing the patient inclusion process.

All adult (>18 years) patients operated on for an intracranial meningioma at the Karolinska University Hospital during a period of 8 years (February 2005–June 2013), were identified. Patients with WHO grade 1 meningioma were excluded. All patients with WHO grade 2 or 3 meningioma at either index or reoperation were included. During the study period, 537 patients were operated on for a WHO grade 1 meningioma and 51 (8.6%) for either a primary or secondary WHO grade 2 or 3 meningioma. All 51 patients with a WHO grade 2 or 3 meningioma diagnosed at either index or reoperation were included in the baseline analysis for this study. Among these 51 patients, 36 underwent their first surgery between 2005–2013, while the remaining 15 were reoperations with the index surgery performed between 1974 and 2003 (Figure 1).

#### 2.2.5. Statistics

The Shapiro–Wilks test was used to evaluate the normality of the data. Parametric continuous data were presented as a mean (standard deviation), non-parametric continuous data as a median (Q1–Q3), and categorical data as numbers (proportion). The Wilcoxon matched-pairs signed-ranks test was used to determine if there was any significant change in Karnofsky Performance Status (KPS) before and after surgery. Tumor control and overall survival were plotted using Kaplan–Meier curves. Uni- and multivariable logistic regression was used to assess potential predictors of tumor growth/recurrence. All analyses were conducted using the statistical software programs R and SPSS. Statistical significance was set at *p* < 0.05.

#### 2.2.6. Ethical Considerations

The study was approved by the National Ethical Review Authority (registration numbers 2020-00192 and 2021-03623) who waived the need for informed consent regarding the retrospective part of the study. Signed informed consent was obtained from each participant in the prospective quality of life part of the study. Data from the Stockholm Public Health Survey was based on individuals who gave informed consent to participate and was anonymized when obtained.

### 2.3. Results

#### 2.3.1. Baseline Data and Tumor Characteristics

In total, 51 patients met the inclusion criteria and were included in the study. Of these, 15 (29%) had undergone at least one prior meningioma resection whereas 7 (47%) were WHO grade 1 at the most recent prior surgery. The median age was 64 years and 51% were female. A total of 84% (n = 43) of the tumors were WHO grade 2 and the remaining 8 (16%) were WHO grade 3 (Table 1). The most common pre-operative symptom was motor deficit (n = 29, 57%) (Supplementary Table S1).

#### 2.3.2. Treatment and Complications

A Simpson grade 1 resection was achieved in 37% of cases, grade 2 in 25%, grade 3 in 3.9%, and grade 4 in 33% (Table 2). A serious postoperative complication (Ibanez grade II-IV) occurred in six (12%) patients, with one patient passing away due to postoperative arrhythmia (Supplementary Table S1).

**Figure 2:**
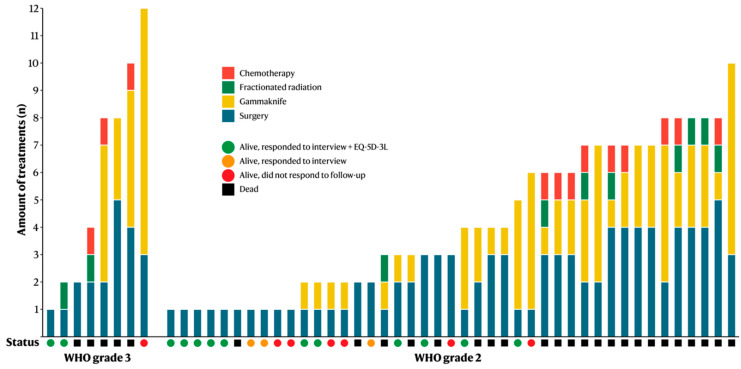
Bar chart showing the total amount of treatment sessions for each individual patient, as well as if the patient responded to the health-related quality of life questionnaire.

Supplementary fractionated RT was performed in 10 (20%) patients, with a total dose of 50–60 Gy. No patient underwent more than one session of fractionated RT. Gamma-knife treatment was performed in 32 (63%) patients, with residual or recurring meningiomas receiving 15–18 Gy to 50% isodose, with a maximum dose of 36–40 Gy. The median number of gamma-knife treatments was 2, although one patient underwent as many as 9 sessions (WHO grade 3, Simpson grade 4). Chemotherapy was administered to 12 (24%) patients (different combinations of Temozolomide, Letrozole, Bevacizumab, and Hydroxikarbamid). The median survival following the start of chemotherapy was 14 months (range 1.9–67 months); none of the patients were alive in 2022 (Figure 2).

#### 2.3.3. Outcomes

**Figure 3:**
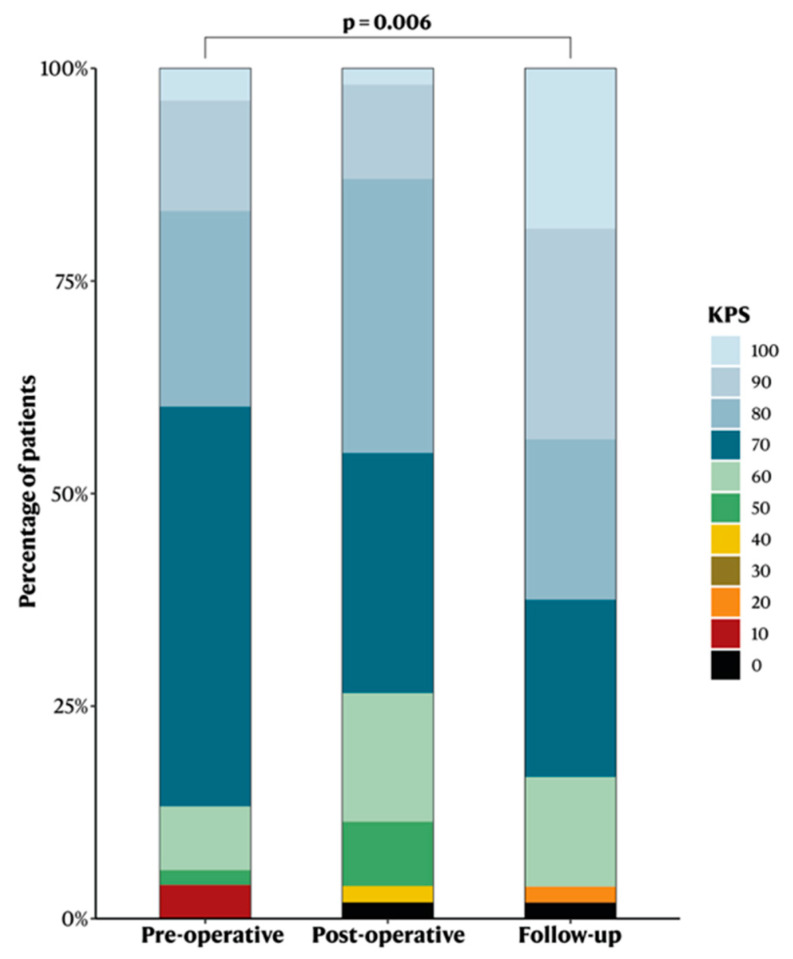
Bar chart showing the Karnofsky Performance Status before surgery, after surgery, and at the 3–6 months postoperative follow-up.

The median pre-operative KPS was 70 (“cares for self but unable to carry on normal activity or to do active work”) (Table 1). Surgery was associated with a significant improvement in KPS (*p* = 0.006). Two patients were unconscious at admission (GCS < 8, KPS 10) but improved to KPS 50 and 60 at discharge, and KPS 70 and 90 at follow-up, respectively (Figure 3).

**Figure 4:**
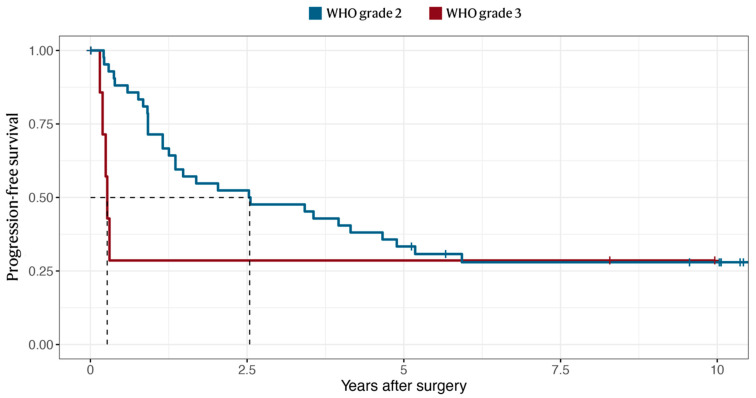
The Kaplan–Meier survival curve of tumor recurrence or growth following surgical resection of WHO grade 2 or 3 meningiomas. The median PFS occurred at 31 months for grade 2 and at 3.4 months for grade 3 meningiomas.

**Figure 5:**
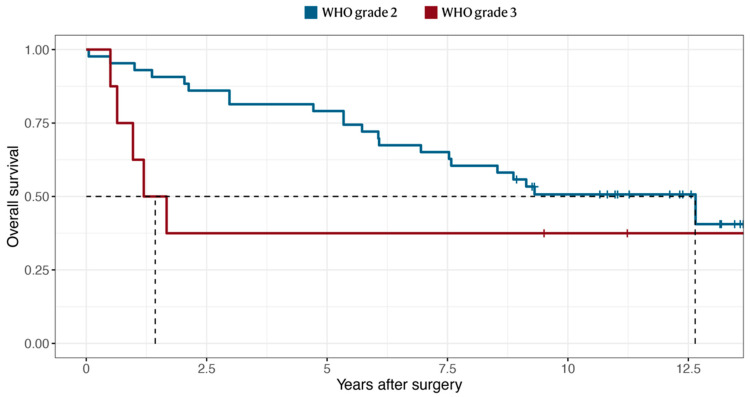
The Kaplan–Meier survival curve of overall survival following surgical resection of the included cohort, stratified by WHO grade. The median OS occurred at 13 years for grade 2 and at 1.4 years for grade 3 meningiomas.

The median radiological follow-up time was 8.1 years, with no patient lost to follow-up. The median progression-free survival was 32 months for WHO grade 2 tumors and 3.4 months for WHO grade 3 (Figure 4). The median survival time (OS) was 13 years for WHO grade 2 tumors, and 1.4 years for WHO grade 3 (Figure 5), conferring 5- and 10-year survival rates of 73% and 45% for the entire cohort, respectively (Table 2). In comparison, the 10-year survival rate for patients who had not undergone any tumor resection prior to the study period (2005–2013) was 60% for WHO grade 2 and 33% for WHO grade 3 tumors (Supplementary Table S2). The median PFS was shorter in tumors classified as “high-risk” according to Rogers et al. [12] (10 vs. 46 months, *p* = 0.018) (Supplementary Table S3).

#### 2.3.4. Predictors of Tumor Growth/Recurrence

In the univariable logistic regression predicting tumor recurrence or growth, a significant association was seen for the MIB-1-index (*p* = 0.018, OR 1.12), but not for female sex, age, ASA class, reoperation, hotspots, or partial tumor resection. The MIB-1-index also showed independent risk association after adjusting for these parameters in the multivariable model (*p* = 0.047, (Table 3).

### 2.4. Discussion: Long-Term Follow-Up and Outcome

Motivated by the poor outcomes for patients with WHO grade 2 and 3 meningiomas, this study sought to assess the long-term clinical and radiological outcomes in a consecutive cohort of 51 patients surgically treated for a WHO grade 2 or 3 meningiomas. The aim was to identify factors influencing the outcome, both in terms of survival and health-related quality of life (HRQoL), to help guide future treatments of these patients. The analyses included baseline data, postoperative complications, supplementary treatments, predictors of recurrence, PFS, OS, and cause of death.

In the univariable logistic regression analysis, the MIB-1-index showed a significant association with tumor recurrence or growth (*p* = 0.018, OR 1.12) and remained an independent predictor even after confounder adjustment (*p* = 0.047). Surprisingly, the extent of tumor resection showed no significant association with PFS. These findings are, in part, different from a recent report where, in addition to the MIB-1-index, a larger tumor size, older age, and necrosis on MRI were identified as predictors of tumor recurrence [28]. Interestingly, approximately 25% of grade 2 and grade 3 tumors remained stable without recurrences over 10 years of follow-up. These discrepancies reflect the extensive heterogeneity, especially among grade 2 patients. Clearly, short-term recurrence reflects the expected behavior of a grade 2 or 3 tumor, while those without progression or recurrence after 10 years behave like grade 1 tumors and may have been cured. Recent papers have indicated a possibility of improved delineation of phenotypes with the addition of molecular markers and a novel molecular classification has been proposed by Driver et al. [29] and Sahm et al. [30] For example, it has been shown that RNA profiles in WHO grade 1 meningiomas differ between tumors undergoing malignant transformation and those that do not [31], and that TERT-promotor mutations mark a significant worsening of the prognosis [32]. In line with this, the WHO 2021 grading has identified TERT-promotor mutated meningiomas as grade 3 tumors.

#### 2.4.1. Overall Survival and Recurrences

We identified a median OS of 13 years for WHO grade 2 and 1.4 years for grade 3 meningiomas. When dichotomized into low- or high-risk [12], low-risk meningiomas had a significantly longer PFS (46 vs. 10 months; *p* = 0.018). Barthelemy et al. studied a large cohort of 3611 grade 2 meningiomas from the National Cancer Database treated between 2008 and 2012 and considered their 78% 5-year survival to be acceptable [33]. A systematic review by Kaur et al. [34] reported a 67.5% 5-year survival after RT in grade 2 meningiomas. The overall 5-year survival of 79% for grade 2 in our cohort is in line with these findings. In grade 3 however, rapid recurrence and short OS were observed in accordance with previously published data [35,36,37].

Of note, previous studies did not address the causes of death. We found that meningioma was the cause of death in 93% of patients who died during the follow-up period.

#### 2.4.2. Radiotherapy and SRS

The evidence for radiotherapy is inconclusive but treatment guidelines typically recommend adjuvant radiotherapy (RT) after gross total removal for grade 2, grade 3, and “high-risk” meningiomas [12]. SRS is typically recommended for small residual or recurrent tumors [24]. Several studies have shown that RT improves PFS but not OS [38,39]. Rogers et al. [12] reported a 59% 3-year PFS and 69% local control after RT for high-risk meningiomas, indicating that RT may be beneficial in these patients. Reported outcomes in the literature are often derived from observational studies or retrospective comparisons with unclear criteria for RT. In contrast, patients in our cohort did not receive routine RT after surgery for a grade 2 or 3 meningioma. Time to recurrence was shorter and tumor control worse in our patients than in patients who received RT after surgery in the published literature [38,39]. However, long-term follow-ups typically show that meningiomas recur despite RT, indicating that the benefit is the delay of recurrence rather than the cure. Interestingly, our numbers for overall survival at 5 and 10 years seem better than literature data for grade 2 meningiomas; unfortunately, our cohort of grade 3 meningiomas is too small for conclusions on this matter. While PFS is an important measure of treatment efficacy, OS in combination with HRQoL is a more relevant measure of benefit to the patient. Two ongoing randomized control studies are investigating the effect of adding adjuvant radiotherapy to gross total resections in patients with atypical meningiomas. However, these studies are designed for analyzing PFS rather than OS [40,41]. RT acts through the induction of DNA damage and malignant cells are more susceptible. However, meningiomas are relatively resistant to radiation [42]. On a speculative note, this may explain why RT improves PFS but not OS and why SRS only improves OS. RT is typically given in a single series of treatments and will affect the actively proliferating tumor cells, but slowly proliferating cells can survive and drive continued tumor growth at a later time point. Conversely, SRS, when used as an alternative to renewed surgery, is directed towards growing tumor nodules and given at much greater doses with a more pronounced effect on all cells in the target volume, resulting in local tumor control. In addition, SRS can be repeated to treat new recurrences, which are often seen outside of previously treated areas [24,43]. Radiation for residual or recurrent meningiomas should be differentiated from preventive therapies after gross total removal. Radiosurgery and hadron therapies allow for more targeted treatment plans to reduce radiation-induced tissue damage. In comparison, RT may cause considerable neurological impairments and handicaps [12], and increase the risks associated with future surgeries through its negative impact on tissues and wound healing [44].

The reported values for local control are 60% at 5 years for proton and 63–95% for carbon ion therapy at 2 years [45]. In our data, only 10 patients received RT while 32 received SRS for a residual or recurrent tumor, since we primarily use SRS for tumor control and RT as the last line rescue therapy, therefore, no comparison could be made. Nonetheless, our strategy of withholding RT and using additional surgery or SRS at our department seems to result in OS data similar to, or better than, those reported for routine RT. In this context, it is important to note that SRS is associated with fewer treatment-related side-effects. Timing of SRS after surgery seems to be of importance, with better results (longer PFS) reported when SRS is given as an adjuvant directly after surgical resection, compared to when it is given as salvage treatment when a progression is observed [24]. Adverse radiation effects (ARE) after SRS are more common in patients treated for atypical meningiomas compared to patients with low-grade meningiomas—reported in 18% of patients with atypical meningiomas and usually emerging within the first 6 months after treatment. While in-field tumor control is 84%, marginal and distant tumor progression are still frequent [24].

We conclude that SRS is useful for the management of grade 2 and 3 meningiomas but did not provide cures.

#### 2.4.3. Chemotherapy

The impact of chemotherapy as supplementary treatment at the study center remains unclear as it was used as a last resort. All treated patients were dead at the time of the structured interview in 2022, and the median survival after starting treatment was 14 months.

### 2.5. Conclusions: Part 1

Taken together, we confirm dismal prognoses, with 93% of the mortality attributed to meningiomas. Yet, the cohort was heterogenous, and a large subgroup comprising both grades 2 and 3 was alive at 10 years follow-up after one operation or operations supplemented with radiosurgery, suggesting that a cure is possible. In contrast, fractionated radiotherapy and chemotherapy had little benefit when introduced for recurrent or progressive diseases.

## 3. Part 2, Health-Related Quality of Life and Return to Work

### 3.1. Introduction

Currently, different combinations of surgery, radiation, and chemotherapy are used to combat grade 2 and 3 meningiomas. However, there are no internationally accepted treatment guidelines. In contrast to the treatment of grade 1 meningiomas, where a single surgery is often sufficient, higher-grade meningiomas frequently recur and require a series of treatments during the lifespan of the patient. Thus, it is of great importance to understand the effects of treatment choices on outcomes as well as the patients’ health-related quality of life (HRQoL).

HRQoL measures can be viewed as self-perceived health [46]. Only a few studies have assessed HRQoL in patients with intracranial meningiomas. When HRQoL was evaluated in grade 1 patients more than ten years after surgery, considerable limitations were found in cognitive, emotional, and social functioning [47]. Determinants for poor HRQoL or impaired cognitive function in patients with WHO grade 1 or 2 meningiomas, studied nine years after surgery, included surgical complications, reoperations, RT, and a large tumor diameter [48]. Studies focusing on HRQoL and return to work in grade 2 and 3 meningiomas are lacking. A nationwide Swedish study on patterns of sick leave and return to work after surgery for cranial meningiomas of any grade showed a decline in the number of patients working full-time, from 79% before surgery to 49% after one year. Two years after surgery, there was a slight recovery to 57%. In comparison, the full-time employment rate for matched controls was 84–86% [49].

In this part of the study, HRQoL and return to work among the patients alive in 2022 were systematically studied.

### 3.2. Materials and Methods

#### 3.2.1. Samples

##### Meningioma Sample

Among the included 51 patients, 23 were still alive in 2022 and were contacted with a request for participation in a follow-up quality of life study. In total, 5 patients declined to participate or did not respond. Thus, 18 patients (15 WHO grade 2 and 3 WHO grade 3) were included in this part of the study, (78% of eligible patients; Figure 1). However, 6 patients submitted incomplete questionnaires.

##### General Population Sample for Comparison of EQ-5D-3L Data

To elicit a comparative general population sample for EQ-5D-3L data, the Stockholm Public Health Survey 2006 was used. It is a cross-sectional survey of a representative sample of the general population in Stockholm County where a self-reported postal questionnaire, including the EQ-5D-3L instrument, was sent to 57,000 adults, with a response rate of 61%. For each of the 13 meningioma patients who answered the EQ-5D-3L, 3 control subjects were randomly selected and individually matched by sex and age using the statistical program SPSS (Version 25, IBM, Armonk, NY, USA). Thus, the anonymized raw data of 39 individuals were included to represent the general population sample.

#### 3.2.2. HRQoL Measurements

##### General Aspects

For the multifactorial analysis of HRQoL a minimum of four aspects are needed: physical-, psychological- and social health or functioning and disease/treatment-related symptoms along with an additional global measure of health status [50]. It is generally agreed that HRQoL is a subjective parameter that can only be assessed individually, and proxy assessments should only be used if the person is unable to make a coherent response [51].

##### EQ-5D-3L

The EQ-5D-3L is a generic HRQoL measure and consists of two parts: a descriptive system where the respondents classify their health in five dimensions (mobility, self-care, usual activities, pain/discomfort, and anxiety/depression) within three severity levels (no problems, moderate problems, or severe problems), and a visual analog scale (EQ_VAS_) [52]. The response value of each dimension in the descriptive system is combined into a 5-digit value representing a corresponding health profile, which can be indexed into a single overall HRQoL value, the EQ-5D_index_, where 0 represents dead and 1 represents full health. Five dimensions within three answering levels give a total of 243 health profiles that can be elicited from the descriptive system. In this study, the United Kingdom (UK) value set was used to calculate the EQ-5D_index_ [53]. On the EQ_VAS_, the respondents rate their current health between 0 (worst imaginable health) and 100 (best imaginable health). The recall period was the day of completion of the questionnaire.

##### Functional Assessment of Cancer Therapy-Brain (FACT-Br)

FACT-Br was developed as a revalidation of the general version of FACT and a subscale for primary brain cancer patients by Weitzner et al. [54]. FACT-Br includes a total of 50 items on a 5-point Likert scale within the following subscale domains: physical well-being (PWB, 7 items), social/family well-being (SWB, 7 items), emotional well-being (EWB, 6 items), functional well-being (FWB, 7 items), and a brain cancer subscale (BrC, 23 items). Each subscale is summarized and produces a subscale score [55]. The summarized subscale scores can be combined into different total scores: a FACT-G total score (summarizes PWB, SWB, EWB, and FWB, values ranging from 0–108) and a FACT-Br total score (summarizes all subscale scores, values ranging from 0–200). A higher score on all subscales and total scales indicates a better quality of life or function. The recall period was the last 7 days.

##### Hospital Anxiety and Depression Scale (HADS)

HADS is a 14-item measure for detecting states of anxiety and depression, constructed by Zigmond and Snaith [56]. The items are divided into two subscales, one for depression (HADS-D) and one for anxiety (HADS-A), answered on a Likert scale and with a recall period of the last week. Each sub-score ranges from 0–21, where higher scores indicate higher severity [56]. The total scale (HADS-T) can also be considered a unidimensional scale of mental distress [57]. Different cut-off scores may be used depending on patient groups and context. The most frequently used cut-off score is ≥8 on both subscales, indicating anxiety or depression (used in primary care) [58]. But lower cut-off scores have been suggested for cancer patients to avoid under-recognition of cases [59]. Optimal cut-off scores for cancer patients (best trade between sensitivity and specificity) have been calculated to be ≥7 for HADS-A, ≥5 for HADS-D, and ≥13 for HADS-T [60].

##### Structured Interviews

Structured interviews were conducted by the first author, either in a secluded room during a follow-up visit at the department of neurosurgery, or by telephone (according to the patients’ preferences). The interviews were conducted between nine and thirteen years postoperatively (index operation). During the interviews, neurological symptoms, subjective cognitive impairment, return to work, family relationships, and leisure activities were assessed. The questions that were asked were concerning the period before and immediately after surgery, the entire follow-up period, and patients’ current situation. The interviews were descriptive, and no formal scales were used. The first author has more than twenty years of experience working clinically with meningioma patients.

#### 3.2.3. Statistics

The EQ-5D-3L data were compared between the meningioma sample and the general population sample. Descriptive statistics for the demographic and study-specific variables, EQ-5D dimensions, EQ-5D_index_, and EQ_VAS_ were calculated. To analyze differences between groups, Fisher’s exact test and t-test were used. Moderate and severe levels on EQ-5D dimensions were collapsed before performing the chi-square statistics. All analyses were conducted using the statistical software program SPSS. Statistical significance was set at *p* < 0.05.

### 3.3. Results

#### 3.3.1. Comparison of HRQoL Results Measured with EQ-5D-3L between the Meningioma Sample and the General Population Sample

A total of 13 patients (6 men) chose to answer the EQ-5D-3L questionnaire. The median follow-up time was 12 years (IQR= 10–13) after the first meningioma surgery. The median age for both cases and controls was 66 years (IQR= 58–76).

Of the possible 243 health profiles that can be elicited from the descriptive system, 8 profiles were selected in the meningioma sample, in comparison to 11 profiles in the general population sample. Full health (i.e., scoring ‘no problem’ in all five dimensions) was rated by n = 2 (15%) in the meningioma sample and n = 17 (44%) in the general population sample. The best imaginable health (i.e., a score of 100 on EQ VAS) was rated by n = 5 (13%) in the general population sample, whereas the highest EQ VAS score in the meningioma sample was 93.

The meningioma sample rated significantly more problems with anxiety/depression in comparison to the general population sample. No other significant differences were found within the EQ-5D-3L dimensions, EQ-5Dindex, or EQ VAS between the samples (Table 4).

#### 3.3.2. HRQoL Measured with FACT-Br

A total of n = 12 participants (equal numbers of men and women) in the meningioma sample chose to answer the FACT-Br questionnaire, the results were not normally distributed. When comparing central tendency and dispersion with previously published reference samples, the meningioma sample scored similarly to US adult cancer patients on PWB, SWB, EWB, FWB subscales, and FACT-G total score [61], and higher on the FACT-Br subscale and FACT-Br total score in comparison to cerebral meningioma patients without epilepsy (where only mean values were presented) [62] (Table 5).

#### 3.3.3. Anxiety and Depression, Measured with HADS

The same twelve participants that answered FACT-Br in the meningioma sample also chose to answer the HADS questionnaire. When using the cut-off scores for cancer patients suggested by Singer et al. [60], a total of n = 5 (42%) of participants scored above the cut-off point of ≥13 on the HADS-T scale, indicating mental distress. The corresponding numbers for the subscales were n = 6 (50%), scoring above the cut-off point of ≥7 on HADS-A, and n = 4 (33%) above the cut-off point of ≥5 on HADS-D. Table 6 shows the HADS scale results for the meningioma sample and comparable values from a randomized Swedish sample [63].

#### 3.3.4. Results from Structured Interviews

All living patients were invited to a structured interview, of whom 16 chose to participate. Two patients refrained from participating in the interview, due to other health-related reasons, but were overall content following their previous surgery. The median follow-up time from index surgery for these 16 patients was 12 years (range 9.3–15 years). Two patients had undergone a reoperation for tumor recurrence after their index surgery, five underwent supplementary gamma-knife treatment, and one RT (Figure 2). Depending on tumor localization, patients experienced different neurological symptoms, and all but one had focal neurological symptoms. All patients experienced some degree of subjective cognitive impairment (Table 7). When the patients were asked whether they would consider undergoing renewed surgery, the most common response was that they did not have a choice, they felt they had no alternative. Half of the patients stated that their family life had been negatively affected. Four patients experienced affected leisure activities, in terms of loss of interest or difficulties in resuming previous hobbies. A total of 13 patients worked before surgery (Table 7); postoperatively, 12 patients returned to work while 1 patient received an old-age pension.

### 3.4. Discussion, HRQoL, and Return to Work

Recently, systematic reviews have revealed that grade 1 meningiomas, although generally considered benign, cause a substantially reduced quality of life in affected patients compared to a normal population at short-term follow-up [64,65]. Neither long-term effects for patients with grade 2 and 3 meningiomas have been studied. In fact, only 18 studies on HRQoL in meningiomas could be found [64,65]. The heterogeneous reporting (differing in the use of HRQoL scales, follow-up time, WHO grade, and age at follow-up) in previous studies precludes comparison and interpretation of our HRQoL data in an international context. HRQoL results are difficult to generalize since the frequency of health problems increases with age, negatively impacting the EQ-5D_index_ values [66,67]. The increased problems reported in higher ages correspond with results from Timmer et al. [68], who assessed HRQoL 3.8 years after meningioma surgery in an elderly sample (median age 67 years) using SF-36 and found declined function in several HRQoL dimensions among the older age groups in comparison with those 55–59 years of age.

Surprisingly, the meningioma sample did not differ from the matched general population sample in the EQ-5D_index_ (0.74 versus 0.77). Previous studies have shown that meningioma patients generally report a worse HRQoL than healthy controls [65]. By necessity, the patients that were available for analysis of long-term HRQoL and interviews were surviving patients with few neurological deficits. The lack of a significant difference in the EQ-5D_index_ between patients and controls could thus reflect this selection bias. In line with this, epilepsy, which is strongly associated with worse health after meningioma surgery [47,62], occurred in 41% of the whole cohort, but not in any of the interviewed patients. Additional reasons for the non-significant results on the EQ-5D_index_ could be that the randomized general population sample also reported high numbers of health problems due to high age or comorbidities [69], or insufficient sample sizes [70].

The EQ-5D-3L questionnaire showed significantly higher scores for the anxiety and depression dimension, where meningioma patients scored higher for moderate or severe problems than the matched general population sample (*p* = 0.003). This high perceived anxiety/depression was also visible in the HADS scoring. The overall HADS scores were higher than reported by a randomized sample of the Swedish population [63]. Our patients also had higher scores for anxiety and depression than a Norwegian population of elderly patients with grade 1 meningioma [71]. The Norwegian study targeted WHO grade 1 patients who were followed up 6 months after surgery, in contrast to our sample of patients with more aggressive WHO grades 2 or 3 and who have undergone multiple treatments and tumor recurrences during an extended period of time.

The meningioma patients in our sample scored similarly to the general population sample on the EQ_VAS_ (68 versus 72). One reason for not presenting the EQ_VAS_ as a general perception of HRQoL in studies may be that the measure is highly susceptible to response shift, i.e., a changed evaluation of a target construct as a result of the person’s internal standards of measurement or scale recalibration. For instance, patients may do upward- or downward comparisons (compare themselves with someone better or worse off), which sets the standard for their rating. To assess a possible response shift and its direction, comparisons of a baseline and a retrospective measure are needed [72]. Few studies on the HRQoL using the EQ-5D-3L after meningioma surgery report EQ_VAS_ values, thus the patients’ subjective perception of their current health is missing. However, interviews allowed further analyses of patients’ subjective health.

The HRQoL is also negatively affected by tumor recurrence which has a higher frequency than, for instance, spinal meningiomas where patients have an HRQoL similar to the general population [73,74,75]. The reported values in the different subscales of FACT-BR did not significantly differ from adult cancer patients or grade 1 meningiomas [62].

All interviewed patients had some degree of perceived cognitive impairment. This is in contrast to previous findings on grade 1 meningiomas where, except for working memory, no differences in memory, visuo-constructive abilities, or executive functions could be seen compared to preoperative assessment, while an improvement in attentional functions was observed at 9 months after surgery [76]. On the other hand, Hendrix et al. [77] showed that grade 1 meningioma patients had significant cognitive impairments within the areas of perceptual speed, executive functions, short-term memory, and verbal fluency, pre- and postoperatively when compared to matched controls. However, none of their meningioma patients experienced cognitive deterioration after surgery. In comparison to our results, van der Vossen et al. [78] reported a smaller proportion (23%, n = 31) of mostly meningioma grade 1 patients having subjective cognitive complaints about 30 months after surgery. Importantly, cognitive function declines during senescence. Most of the patients evaluated with questionnaires and interviews were above 65 years of age. A decline in cognitive function has been shown to occur in meningioma patients from the age of 55 [79].

Despite reporting neurological deficits and impaired cognition, all interviewed patients had been able to return to work. Of the 13 patients working before surgery, all but one, who reached old-age pension, returned to work. This contrasts with previous reports on large populations that indicate that 17–33% of the patients operated on for meningioma were unable to return to work [80,81].

### 3.5. Conclusions: Part 2

From a long-term follow-up perspective, our patients report an HRQoL comparable to that of meningioma grade 1 patients. The patients suffer some cognitive impairments and report emotional problems such as anxiety, depression, and fatigue as common symptoms after surgery [47,78,80,82]. It appears that the presence of a potentially chronic brain tumor may cause some of these problems since asymptomatic patients with meningioma who have not been operated on also display a high incidence of psychiatric complaints affecting their life [83,84]. Despite suffering from a chronic disease and in contrast to previous reports, all patients working before surgery returned to work after surgery.

## 4. Limitations

This study carries limitations inherent to its retrospective design. The study is also limited by the small number of patients fulfilling criteria for grades 2 and 3 with the 2007 classification. However, the use of that classification allows comparisons to similar cohorts [7] but will make external validity for the WHO 2021 grading weak. The presented PFS and OS for eight patients with grade 2 or 3 tumors operated on and diagnosed prior to the study period were based on the time from the first surgery performed within the study period. Thus, this does not represent the time from their initial surgery. The explanation for this is that all patients were identified from records within the study period. This is likely to result in a bias towards shorter PFS and OS. However, since we cannot access the patient charts for all patients operated on before the study period, there is no way to collect the true survival times. Another limitation is that no preoperative HRQoL data were available to allow comparison to our long-term follow-up findings. The sample size for the HRQoL assessment was also small and based solely on survivors.

## 5. Conclusions

Patients with grade 2 and 3 meningiomas have a chronic disease requiring multiple surgeries and often supplementary treatments. Patients with high-risk tumors have a significantly shorter PFS compared to low-risk tumors. The MIB-1-index is an independent predictor of tumor recurrence or growth. Long-term surviving patients have a good HRQoL compared to the general population, with the exception of some degree of cognitive impairment, anxiety, and depression. This subgroup of patients needs fewer treatments, have a more benign clinical course, and returns to work at a high frequency.

## Figures and Tables

**Table 1:** Baseline data.

Variable	All Patients (n = 51)	WHO Grade 2 (n = 43)	WHO Grade 3 (n = 8)
Female sex	26 (51%)	22 (51%)	4 (50%)
Age	64 (52–70) range: 30–79	64 (52–70) range: 30–79	68 (61–72) range: 40–77
KPS	70 (70–80) range: 10–100	70 (70–80) range: 10–100	70 (60–73) range: 10–90
ASA class	3.0 (2.0–3.0) range: 2–5	3.0 (2.0–3.0) range: 2–5	3.0 (2.8–3.0) range: 2–3
Prior meningioma resection	15 (29%)	10 (23%)	5 (62%)
Years since last resection	9.4 (5.7–12) range: 3.0–24	8.8 (5.3–12) range: 3.0–24	9.4 (8.3–11) range: 5.5–13
WHO grade at last resection			
WHO grade 1	7 (47%)	5 (50%)	2 (40%)
WHO grade 2	7 (47%)	5 (50%)	2 (40%)
WHO grade 3	1 (6.7%)	0 (0%)	1 (20%)

Data are presented as median (Q1–Q3) or number (proportion). Abbreviations: ASA = American Society of Anesthesiologists; KPS = Karnofsky Performance Status; WHO = World Health Organization.

**Table 2:** Treatment and outcome data.

Variable	All Patients (n = 51)	WHO Grade 2 (n = 43)	WHO Grade 3 (n = 8)
**Simpson grade**			
Simpson 1	19 (37%)	17 (40%)	2 (25%)
Simpson 2	13 (25%)	12 (28%)	1 (13%)
Simpson 3	2 (3.9%)	1 (2.3%)	1 (13%)
Simpson 4	17 (33%)	13 (30%)	4 (50%)
**Tumor characteristics**			
MIB-1-index	15 (8.0–20) (2 missing)	12 (8.0–20)	23 (16–29) (2 missing)
**Supplementary treatment**			
Fractionated radiotherapy	10 (20%)	8 (19%)	2 (25%)
Gamma-knife treatment	32 (63%)	28 (65%)	4 (50%)
Treatment sessions	2 (1–4) range: 1–9	2 (1–3) range: 1–7	2 (1–3) range: 1–9
Chemotherapy	12 (24%)	9 (21%)	3 (28%)
**Tumor control**			
Radiological follow-up time (years)	8.1 (3.4–10)	8.8 (5.0–10)	1.3 (0.7–8.7)
Tumor growth/recurrence	35 (69%)	30 (70%)	5 (63%)
Median progression-free survival (months)	30	31	3.4
Simpson grade 1–2	42	43	2.3
Simpson grade 3–4	11	13	3.7
**Survival**			
Dead	28 (55%)	23 (53%)	5 (62%)
Death due to meningioma	26 (93%)	21 (91%)	5 (100%)
5-year survival	37 (73%)	34 (79%)	3 (38%)
10-year survival	21 (45%) (4 censored *)	19 (48%) (3 censored *)	2 (29%) (1 censored *)
Median survival (years)	9.3	13	1.4
Simpson grade 1–2	13	13	1.2
Simpson grade 3–4	8.9	9.0	1.3

Data are presented as median (Q1–Q3) or number (proportion). * Censored as the follow-up time was less than 10 years [12].

**Table 3:** Predictors of tumor recurrence/growth.

Variable	Univariable OR (95% CI)	Univariable *p*-Value	Multivariable *p*-Value
Female sex	1.06 (0.32–3.50)	0.925	0.854
Age (years)	1.05 (1.00–1.11)	0.067	0.289
ASA class	0.54 (0.15–1.57)	0.274	0.225
MIB-1-index	1.12 (1.03–1.23)	**0.018**	**0.047**
Reoperation	2.26 (0.59–11.3)	0.266	0.996
Hotspots	2.20 (0.54–8.83)	0.262	0.935
Partial resection (Simpson grade 3–4)	2.25 (0.64–9.32)	0.098	0.306

Abbreviations: ASA = American Society of Anesthesiologists; cm = centimeter; CI = confidence interval; OR = odds ratio. Bold text indicates a statistically significant correlation (p < 0.05).

**Table 4:** Percentage (number) of respondents reporting no, moderate, and severe problems in EQ-5D dimensions, EQ-5D index, and EQ VAS, meningioma sample and general population sample.

EQ-5D Dimensions	Meningioma Sample, n = 13		General Population Sample, n = 39		*p* ^1,2^
	%	(n)	%	(n)	
**Mobility**					0.709
No problems	85	(11)	71	(30)	
Moderate problems	15	(2)	23	(9)	
Severe problems	0.0	(0)	0.0	(0)	
**Self-care**					1.000
No problems	92	(12)	95	(37)	
Moderate problems	0.0	(0)	5.1	(2)	
Severe problems	7.7	(1)	0.0	(0)	
**Usual activities**					1.000
No problems	77	(10)	77	(30)	
Moderate problems	15	(2)	21	(8)	
Severe problems	7.7	(1)	2.6	(1)	
**Pain/discomfort**					1.000
No problems	38	(5)	44	(17)	
Moderate problems	62	(8)	44	(17)	
Severe problems	0.0	(0)	13	(5)	
**Anxiety/depression**					0.003
No problems	23	(3)	72	(28)	
Moderate problems	77	(10)	28	(11)	
Severe problems	0.0	(0)	0.0	(0)	
EQ-5D_index_ mean (±SD)	0.74 (±0.23)		0.77 (±0.29)		0.777
EQ_VAS_ mean (±SD)	67.8 (±24.9)		72.1 (±24.1)		0.582

^1^ Differences between the meningioma sample and general population sample. ^2^ Moderate and severe levels in EQ-5D dimensions collapsed before Chi-square analysis. Bold text indicates a statistically significant correlation (*p* < 0.05).

**Table 5:** FACT-Br scale results, meningioma sample, and reference groups.

Scale (Score Range)	Meningioma Sample, n = 12			Reference Groups (Central Tendency and Dispersion)		
	Range	Median	(IQR)	Median	(IQR)	
PWB subscale (0–28)	13–28	23	(20–26)	23	(18–26)	Adult patients, different cancer sites, n = 2236 [61]
SWB subscale (0–28)	6–28	23	(18–26)	23	(19–26)	
EWB subscale (0–24)	6–24	19	(18–23)	20	(16–22)	
FWB subscale (0–28)	3–26	20	(12–25)	20	(14–25)	
FACT-G total (0–108)	31–105	87	(66–95)	83	(70–95)	
				Mean	(SD)	
BrC subscale (0–92)	29–89	74	(59–79)	60	(14)	Meningioma patients, n = 109 [62]
FACT-Br total score (0–200)	60–191	162	(126–173)	146	(30)	

Abbreviations: BrC = brain cancer; EWB = emotional well-being; FWB = functional well-being; FACT-Br = Functional Assessment of Cancer Therapy-Brain; SWB = social/family well-being; PWB = physical well-being.

**Table 6:** HADS subscale and total scale scores, meningioma sample, and reference group.

Scale	Meningioma Sample n = 12			Randomized Swedish Sample, n = 624 [63]	
	Range	Median	(IQR)	Median	(IQR)
HADS-A	0–16	5.5	(2.3–9.0)	4.0	(2.0–7.0)
HADS-D	1–15	2.0	(1.3–8.8)	3.0	(1.0–6.0)
HADS-T	1–31	10	(4.0–19)	7.0	(4.0–12)

Abbreviations: HADS Hospital Anxiety and Depression Scale.

**Table 7:** Postoperative, subjective cognitive impairment, employment status, and return to work.

Variable	Patients (n = 16)
Subjective cognitive impairment	
After index surgery	16 (100%)
At the time of interview	
Improved	5 (31%)
Unchanged	9 (56%)
Worse	2 (13%)
Employment status	
Before surgery	
Full time	13 (82%)
Full time sick leave	1 (6.3%)
Sickness pension	1 (6.3%)
Old-age pension	1 (6.3%)
After surgery	
Full time	9 (56%)
Part time	3 (19%)
Sickness pension	2 (13%)
Old-age pension	2 (13%)

Data are presented as numbers (proportion).

## Data Availability

Data are available from the corresponding author upon reasonable request.

## Supplementary Materials

The following supporting information can be downloaded at: https://www.mdpi.com/article/10.3390/cancers14205038/s1: Supplementary Table S1; Pre-operative symptoms and postoperative complications stratified by WHO grade; Supplementary Table S2: Outcome data stratified by if the patients underwent their first surgery before or during the study period (2005–2013); Supplementary Table S3: Outcome data stratified by low- or high-risk tumor and treatment characteristics.

## Abbreviations

ASA	American Society of Anesthesiologists
HRQoL	Health-Related Quality of Life
MRI	Magnetic Resonance Imaging
OR	Odds Ratio
WHO	World Health Organization
PFS	Progression Free Survival
OS	Overall Survival
RT	Radiotherapy
SRS	Stereotactic Radio Surgery
