# Supplementary material for: Long-Term Follow-Up, Treatment Strategies, Functional Outcome, and Health-Related Quality of Life after Surgery for WHO Grade 2 and 3 Intracranial Meningiomas
Source: Cancers (Basel). 2022 Oct 14;14(20):5038. doi: 10.3390/cancers14205038 (PMC9600120; doi:10.3390/cancers14205038)
Supplement: Supplementary file 1 [file cancers-14-05038-s001.zip › cancers-1938030-supplementary.pdf]

Supplementary Table S1. Pre-operative symptoms and postoperative complications stratified by WHO grade.

Variable	All patients (n = 51)	WHO grade 2 (n = 43)	WHO grade 3 (n = 8)
**Pre-operative symptoms**			
Sensory deficit	8 (16%)	6 (14%)	2 (25%)
Motor deficit	29 (57%)	23 (53%)	6 (75%)
Cognitive deficit	21 (41%)	17 (40%)	4 (50%)
Seizures	21 (41%)	16 (37%)	5 (63%)
**Postoperative complications**			
Ibanez grade Ia	15 (29%)	12 (28%)	3 (38%)
Ibanez grade Ib	15 (29%)	13 (30%)	2 (25%)
Ibanez grade IIa	1 (2.0%)	1 (2.3%)	0 (0%)
Ibanez grade IIb	2 (3.9%)	1 (2.3%)	1 (13%)
Ibanez grade IIIa	2 (3.9%)	2 (4.7%)	0 (0%)
Ibanez grade IV	1 (2.0%)	1 (2.3%)	0 (0%)

Data is presented as number (proportion). Abbreviations: KPS = Karnofsky Performance Status

Supplementary Table S2. Outcome data stratified by if the patients underwent their first surgery before or during the study period (2005-2013)

Variable	WHO grade 2		WHO grade 3	
	Before study period (n = 10)	During study period (n = 33)	Before study period (n = 5)	During study period (n = 3)
Median PFS	9.6 months	41 months	3.7 months	2.3 months
5-year PFS	1 (10%)	12 (36%)	1 (20%)	1 (33%)
10-year PFS	0 (0%)	11 (33%)	1 (20%)	1 (33%)
5-year OS	5 (50%)	29 (88%)	2 (40%)	1 (33%)
10-year OS	1 (10%)	18 (60%) (3 missing)	1 (25%) (1 missing)	1 (33%)

Data is presented as median (Q1-Q3) or number (proportion). Abbreviations: OS = overall survival; PFS = progression-free survival. Median survival not available as < 50% of patients had passed away

Supplementary Table S3. Outcome data stratified by low- or high-risk tumor and treatment characteristics

Variable	Low-risk tumor (n = 28)	High-risk tumor * (n = 23)	p-value
Median progression-free survival	46 months	10 months	**0.018**
5-year survival	23 (82%)	14 (61%)	0.090
10-year survival	14 (54%) (2 missing)	7 (33%) (2 missing)	0.160
Median survival	NA**	7.6 years	-

Data is presented as median (Q1-Q3) or number (proportion).
* High-risk defined as any WHO grade 3, WHO grade 2 recurrence, or WHO grade 2 treated with partial resection (Simpson grade 3-4) [12]
** Median survival not available as < 50% of patients had passed away
